# Medical school service regions in Canada: exploring graduate retention rates across the medical education training continuum and into professional practice

**DOI:** 10.1080/10872981.2024.2403805

**Published:** 2024-11-03

**Authors:** Cassandra Barber, Cees van der Vleuten, Saad Chahine

**Affiliations:** a School of Health Professions Education (SHE), Faculty of Health, Medicine and Life Sciences, Maastricht University, Maastricht, The Netherlands; b Department of Educational Development and Research, Faculty of Health, Medicine and Life Sciences, Maastricht University, Maastricht, The Netherlands; c Faculty of Education, Queen’s University, Kingston, Ontario, Canada

**Keywords:** Social accountability, Graduate retention, Quantitative analysis, School service regions, Geography

## Abstract

**Purpose:**

To create medical school service regions and examine national in-region graduate retention patterns across the medical education continuum and into professional practice as one approach to advancing social accountability in medical education.

**Methods:**

Medical school service regions were created in Canada using publicly available data and mapped using Geographic Information System (GIS) software. Population size and density for each service region were calculated using census data. Retrospective data of medical graduates who completed their medical degrees between 2001–2015 (*n* = 19,971) were obtained from a centralized data repository and used to analyze in-region retention rates by medical specialty across the training continuum and five years into professional practice.

**Results:**

Spatial inequities were observed across medical school service regions. Graduate retention patterns also varied across service region groups and medical specialties. Quebec (86.5%) and Ontario (80.4%) had above-average retention rates across the medical education continuum. Family medicine had the highest retention rates from undergraduate to postgraduate training (81.9%), while psychiatry had the highest retention rate across the training continuum and into professional practice (71.2%). The Alberta and British Columbia service region group demonstrated high retention rates across the training continuum and into professional practice and medical specialties, except for retention from undergraduate to postgraduate medical education.

**Conclusion:**

This study highlights the importance of considering both medical specialty and practice location of graduates when planning and retaining the physician workforce. The observed retention patterns among graduates are a critical aspect of addressing societal needs and represent an intermediate step towards achieving health equity. Furthermore, graduate retention patterns serve as an outcome measure for schools to demonstrate their commitment to social accountability. Tracking and monitoring graduate outcomes may lead schools to actively collaborate with government agencies responsible for healthcare policy, which may ultimately improve physician workforce planning and promote more equitable healthcare access.

## Background

Medical schools are expected to be socially accountable to their local communities by directing their education, research, and service activities towards addressing priority health needs [1–3]. This includes determining the appropriate number, mix, and distribution of physicians necessary to meet societal needs [4]. This paper provides a methodological approach for creating medical school service regions and investigates the variation in national graduate retention patterns within these respective regions.

Considerable research has examined the retention patterns of medical graduates internationally [5–13], with most literature focusing on primary care and rural practice settings [14–20]. Despite efforts to encourage physicians to practice in rural regions where needs are often greatest [4,21,22], regional scarcities persist, contributing to overall shortages and the maldistribution of medical specialists [23]. Several factors influence the ultimate location of professional practice, including personal, family, and professional factors; rural upbringing; location of postgraduate training; and medical specialty [16,20]. Medical school characteristics have also been shown to influence graduate specialty choice and practice location [24]. Targeted, socially accountable rural and local admission pathways, as well as curricular exposure to primary care practices/principles and extended community-based training, foster graduates committed to primary care, making them more likely to practice in local communities [5–13,25,26]. Furthermore, rural upbringing and longitudinal rural community-based placements have significantly increased the number of graduates practicing in rural settings [15,27–35].

Beyond the focus on rural practice choice, many studies have documented the association between graduate practice location and the location of postgraduate training [16,20,32–35]. Primary care graduates are often more likely than specialists to practice in the same location as their postgraduate training [19,20]. For example, Koehler et al. [32] conducted a study in the United States examining the likelihood of graduates practicing in the same state where they completed their residency training. The study suggests that graduates with local connections, such as local hometown origin, completing both undergraduate and postgraduate medical training in the same region, being married, and graduating from a primary care medical specialty, were more likely to practice in-state [32]. Additionally, primary care graduates were more likely to practice in the same region where they completed their residency training compared to other hospital-based specialties [32]. Similarly, Seifer et al. [16] examined the relationship between residency training and practice location, identifying personal characteristics, location of medical training, and financial incentives as factors influencing the practice location of physicians. Their findings suggest that general practitioners (i.e., family medicine, internal medicine, and pediatrics physicians) were also more likely to practice in the same state where they completed their residency compared to non-primary care physicians.

The purpose of this study was to introduce a methodological approach for creating medical school service regions and investigate the variation in graduate retention patterns within these respective regions. Understanding these patterns is critical to advance social accountability in medical education and address local healthcare needs effectively. Service regions may be characterized as geographical areas served by a school or ‘intended area(s) of primary responsibility’ (adapted from Singleton et al. [36]. These regions are not intended to act as absolute boundaries but rather as general regions which medical schools are mandated to serve. This evaluative process can serve as a model to better understand school service regions and evaluate graduate retention patterns as an initial step toward advancing social accountability in medical education.

## Methods

### Study Setting

There were 17 medical schools in Canada at the time this study was conducted, with six located in the province of Ontario, four in Quebec, four in the Prairie provinces (Manitoba, Saskatchewan, and Alberta), one in British Columbia, and two in Atlantic Canada (Nova Scotia and Newfoundland & Labrador). All Canadian medical schools engage in some form of distributed medical education and community-based training [37]. In Canada, physicians must complete a three- or four-year Doctor of Medicine degree (MD), followed by two to five plus years of residency training, and pass a series of regulatory licensing examinations prior to entering unsupervised medical practice [38].

### Creating Medical School Service Regions

To create medical school service regions in Canada, federal administrative health region boundaries [39] were used, along with the location of medical schools and information on schools’ distributed campuses, community training sites, health authorities, and rural and regional education and training opportunities. Health regions are established by provincial ministries of health and are used to make healthcare decisions [40].

The process of creating medical school service regions involved several steps. First, health regions were identified using Statistics Canada 2018 Census geographic units [40]. This was followed by grouping the physical location of each medical school by province. Next, the geographic area of primary responsibility that each medical school served was developed using information obtained from institutional websites [41–57], including distributed campuses, community training sites, and rural and regional education and training opportunities. Once this information was collected, each medical school’s geographic area was assigned to a corresponding health region. Finally, the health regions assigned to each respective school’s regions were aggregated to create medical school service region groups (depicted in ‘Supplemental Appendix I’).

### Data Sources

Publicly available digital boundary files for 2018 health regions, compatible with Geographic Information System (GIS) mapping software, were obtained from Statistics Canada website [40]. GIS is an increasingly recognized tool for mapping and spatial analysis with applications to health systems planning [58], and was used to map medical school service regions. Additionally, Statistics Canada’s 2016 Census of Population [59] was also used to provide data on population counts, population density per square kilometer (km^2^), and land area in km^2^ for each health region. Statistics Canada’s Census of Population is a mandatory cross-sectional survey of the population, conducted every five years [60].

To examine graduate retention patterns, data from the Canadian Post-M.D. Education Registry (CAPER) on medical graduates who completed their MD degree in Canada between 2001–2015 (*n* = 19,971) were obtained. CAPER is an initiative of the Association of Faculties of Medicine of Canada (AFMC) and serves as a central data repository for all postgraduate medical residents, fellows, and practicing physicians in Canada [61].

### Analysis

The analyses were conducted based on the primary area of responsibility of medical schools using school location and their broader service areas, as well as health region boundaries.

A medical school service region map was generated using GIS ESRI ArcGIS Desktop 10.8.1. Population counts, land area in km^2^, and population density per km^2^ were calculated for each medical school region by aggregating values obtained from corresponding health regions. Retention rates were calculated based on the proportion of graduates practicing in the same service region where they completed their undergraduate and/or postgraduate medical training. Mean retention values were compared by medical specialty across the training continuum and five years into professional practice.

To ensure the confidentiality and anonymity of both institutions and individual graduates, medical school service regions were aggregated into larger service region groups: Atlantic, Quebec, Ontario, Manitoba & Saskatchewan, and Alberta & British Columbia. This decision, informed through consultations with CAPER, was primarily driven by the need to protect institutional identities and prevent the identification of individual graduates, especially in regions with a low number of practicing specialists. Aggregating data into larger service region groups mitigates these risks while still allowing for comprehensive analyses of national retention rates without compromising confidentiality.

Medical specialty groups were categorized based on six core clinical areas of medical training: family medicine, internal medicine and subspecialties, obstetrics and gynecology and subspecialties, paediatrics and subspecialties, psychiatry and subspecialties, surgical specialties (excluding obstetrics and gynecology). To maintain confidentiality and prevent the identification of smaller, less populated medical specialties in different geographic regions, these core groups were not further disaggregated.

Retention patterns for Canadian medical graduates were calculated for each service region group using the following outcomes: (1) the proportion of graduates who remained in the same service region group for both undergraduate and postgraduate training, (2) the proportion of graduates who practiced in the same service region group where they received their undergraduate and postgraduate medical training, (3) the proportion of graduates who practiced in the same region where they completed their undergraduate training, and (4) the proportion of graduates who practiced in the same service region where they completed their postgraduate training.

Ethics approval was obtained from Maastricht University (Approval No. FHML-REC/2020/101).

## Results

### Medical school service regions

**Figure 1.**
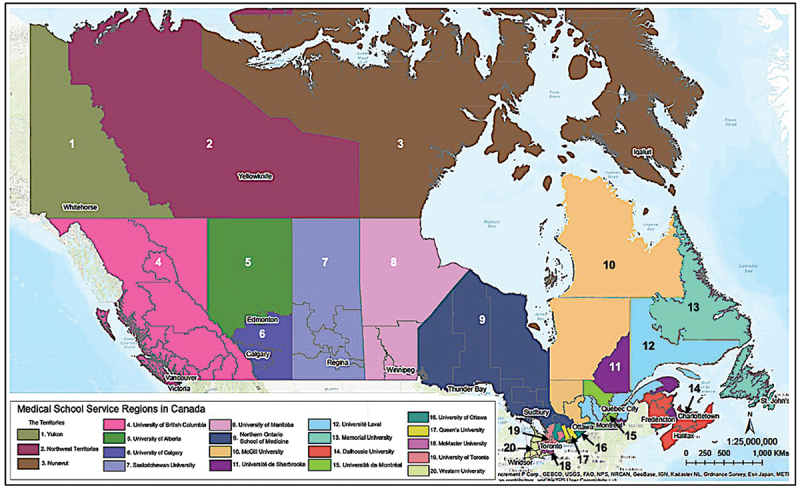
Map showing 17 medical school service regions, comprising of 126 health regions in Canada.

The map, depicted in Figure 1, shows that medical school service regions in Canada were not uniform, revealing spatial inequities in terms of total population, land area in km^2^, and population density/km [2] (shown in Table 1). Ontario served the largest population (13,371,698), with the majority residing in the University of Toronto service region (7,380,462). In contrast, the smallest population resided in Queen’s University service region (354,543). The University of British Columbia was the only medical school located in the province of British Columbia, serving a total population of 4,648,055. The Territories (Nunavut, Yukon, and Northwest Territories) represents 40% of Canada’s land mass and approximately 3% of the total population [62]. While no faculties of medicine reside in the Territories, residency training opportunities have recently become more available, with the responsibility residing in a handful of schools.

**Table 1.** Total population, Land Area in square kilometres (km^2^) and population density per square kilometres (km^2^) by medical school service region in Canada using 2018 health regions boundaries in Canada.

Medical School Service Region	Total Population^a^	Land area (km^2^)^b^	Population Density (km^2^)^c^
*Atlantic Service Region*			
Memorial University of Newfoundland	519,716	370,514	1.4
Dalhousie University	1,604,350	119,990	13.4
**Atlantic Service Region Total**	**2,124,066**	**490,504**	**4.3**
*Quebec Service Region*			
Université de Sherbrooke	1,048,550	141,800	7.4
Université Laval	2,854,653	89,568	31.9
Université de Montreal	3,804,014	51,892	73.3
McGill University	666,400	1,083,392	0.6
**Quebec Service Region Total**	**8,373,617**	**1,366,652**	**6.1**
*Ontario Service Region*			
McMaster University	1,654,902	5,469	302.6
Northern Ontario School of Medicine	882,534	810,295	1.1
University of Ottawa	1,485,332	23,589	63.0
Queen’s University	354,543	13,782	25.7
University of Toronto	7,380,462	23,922	308.5
Western University	1,613,925	29,424	54.9
**Ontario Service Region Total**	**13,371,698**	**906,481**	**14.8**
*Manitoba & Saskatchewan Service Region*			
University of Manitoba	1,278,365	552,371	2.3
University of Saskatchewan	1,133,805	846,387	1.3
**Manitoba & Saskatchewan Service Region Total**	**2,412,170**	**1,398,758**	**1.7**
*Alberta & British Columbia Service Region*			
University of Calgary	2,304,541	195,241	11.8
University of Alberta	1,762,634	445,089	4.0
University of British Columbia	4,648,055	922,503	5.0
**Alberta & British Columbia Service Region Total**	**8,715,230**	**1,562,833**	**5.6**
*Territories*			
Yukon	35,874	474,713	0.1
Northwest Territories	41,786	1,143,794	0.0
Nunavut	35,944	1,877,779	0.0
**Territories Total**	**113,604**	**3,496,286**	**0.0**
Canada	35,151,728	8,965,588.85	3.9

Data was obtained online from Statistics Canada 2016 Census of the Population [60].

We analyzed 19,971 practicing physicians in Canada who graduated with a Canadian M.D. between 2001 and 2015. Descriptive statistics across the training continuum and into professional practice by medical specialty were categorized according to medical school service region groups (depicted in Table 2). Most graduates received their undergraduate (UME) and postgraduate medical education (PME) training in the Ontario service region group (UME = 34.6%, PGME = 38.0%) or Quebec service region group (UME = 31.8%, PME = 29.3%).

**Table 2.** Descriptive statistics of practicing medical graduates in Canada (2001-2015).

Variable Names	No. (%) out of a total of n = 19,971
*Undergraduate Medical Education Service Region Groups*	
Atlantic Region	1,391 (7.0)
Quebec Region	6,354 (31.8)
Ontario Region	6,915 (34.6)
Manitoba & Saskatchewan Region	1,378 (6.9)
Alberta & British Columbia Region	3,933 (19.7)
*Postgraduate Medical Education Service Region Groups*	
Atlantic Region	1,100 (5.5)
Quebec Region	5,854 (29.3)
Ontario Region	7,590 (38.0)
Manitoba & Saskatchewan Region	972 (4.9)
Alberta & British Columbia Region	4,455 (22.3)
*Year 5 Practice Location Service Region Groups*	
Atlantic Region	871 (4.4)
Quebec Region	4,102 (20.5)
Ontario Region	5,156 (25.8)
Manitoba & Saskatchewan Region	730 (3.7)
Alberta & British Columbia Region	3,540 (17.7)
Territories	76 (0.4)
*Medical Specialty*	
Family Medicine	10,894 (54.5)
Internal Medicine & Subspecialties	3,170 (15.9)
OB‎/Gyn & Subspecialties	754 (3.8)
Paediatrics & Subspecialties	1,177 (5.9)
Psychiatry & Subspecialties	1,197 (6.0)
Surgical Specialities (excl. OB/Gyn)	2,779 (13.9)

Note. Data was obtained online from Statistics Canada 2016 Census of the Population.^60^

Approximately 30% of all year-5 practice location data were missing from our sample, primarily observed in 2013, when the file provided by the Canadian Medical Association was missing a significant proportion of postal codes. However, of the 14,475 graduates, one-quarter were practicing in the Ontario service region group (25.8%) five years into professional practice, followed by the Quebec service region group (20.5%), Alberta & British Columbia service region group (17.7%), the Atlantic service region group (4.4%), Manitoba & Saskatchewan service region group (3.7%), and only 0.4% of graduates were practicing in the Territories. In terms of medical specialty, half of all graduates in our sample were family physicians (54.5%), followed by internists and subspecialties (15.9%), surgical specialists (13.9%), psychiatrists (6.0%), paediatricians (5.9%), and the remaining 3.8% practiced in obstetrics and gynecology.

### Retention patterns

Graduate retention patterns for Canadian medical school service region groups across the training continuum and five years into professional practice by medical specialty are depicted in Table 3. Our findings suggest that graduate retention patterns varied across the training continuum and into professional practice, depending on the service region group and medical specialty.

**Table 3.** In-service region group retention by medical specialty (*n* = 19,971).

Medical Specialty & Service Region Group	UGME & PGME^a^			UGME, PGME & Year 5 Practice Region^b^			UGME & Year 5 Practice Region^c^			PGME & Year 5 Practice Region^d^		
	No. of Medical Graduates	Retention Rate (%)	95% CI^e^	No. of Medical Graduates	Retention Rate (%)	95% CI	No. of Medical Graduates	Retention Rate (%)	95% CI	No. of Medical Graduates	Retention Rate (%)	95% CI
*All Specialties*												
Manitoba & Saskatchewan	666	48.3	45.7, 50.9**	390	58.6	54.9, 62.3*	156	21.9	18.9, 24.9	90	29.4	24.3, 34.5
Atlantic	685	49.2	46.6, 51.8**	426	62.2	58.6, 65.8*	146	20.7	17.7, 23.7	118	28.4	24.1, 32.7
Alberta & British Columbia	2,827	71.9	70.5, 73.3*	1,926	68.1	66.4, 69.8*	362	32.7	29.9, 35.5	793	48.7	46.3, 51.1**
Ontario	5,562	80.4	79.5, 81.3*	3,818	68.6	67.4, 69.8**	293	21.7	19.5, 23.9	782	38.6	36.5, 40.7**
Quebec	5,496	86.5	85.7, 87.3*	3,686	67.1	65.9, 68.3**	224	26.1	23.2, 29.0	108	30.2	25.4, 35.0**
**National Avg.**	**15,236**	**76.3**	**75.7, 76.9**	**10,246**	**67.2**	**66.4, 68.0**	**1,181**	**24.9**	23.7, 26.1	**1,891**	**39.9**	**38.5, 41.3**
*Family Medicine*												
Manitoba & Saskatchewan	421	58.7	55.1, 62.3*	245	58.2	53.5, 62.9	53	17.9	13.5, 22.3	36	27.7	20, 35.4
Atlantic	458	61.2	57.7, 64.7*	288	62.9	58.5, 67.3	48	16.6	12.3, 20.9	73	32.0	25.9, 38.1
Alberta & British Columbia	1,623	77.4	75.6, 79.2*	1,100	67.8	65.5, 70.1**	158	33.3	29.1, 37.5	388	54.8	51.1, 58.5*
Ontario	2,993	81.6	80.3, 82.9*	2,131	71.2	69.6, 72.8*	140	20.8	17.7, 23.9	356	47.8	44.2, 51.4*
Quebec	3,422	93.4	92.6, 94.2*	2,275	66.5	64.9, 68.1**	31	12.8	8.6, 17	47	28.3	21.4, 35.2
**Family Medicine Avg.**	**8,917**	**81.9**	**81.2, 82.6**	**6,039**	**67.7**	**66.7, 68.7**	**430**	**21.8**	**20, 23.6**	**900**	**45.5**	**43.3, 47.7**
*Internal Medicine & Subspecialties*												
Manitoba & Saskatchewan	59	27.7	21.7, 33.7**	32	54.2	41.5, 66.9	29	18.8	12.6, 25.0	14	37.8	22.2, 53.4
Atlantic	63	31.7	25.2, 38.2**	40	63.5	51.6, 75.4	32	23.5	16.4, 30.6	9	30.0	13.6, 46.4
Alberta & British Columbia	430	68.1	64.5, 71.7*	290	67.4	63.0, 71.8	59	29.4	23.1, 35.7	163	48.2	42.9, 53.5**
Ontario	924	80.7	78.4, 83.0*	652	70.6	67.7, 73.5**	42	19.0	13.8, 24.2	140	32.6	28.2, 37
Quebec	793	80.8	78.3, 83.3*	533	67.2	63.9, 70.5	51	27.0	20.7, 33.3	25	37.9	26.2, 49.6
**Internal Medicine & Subspecialties Avg.**	**2,269**	**71.6**	**70.0, 73.2**	**1,547**	**68.2**	**66.3, 70.1**	**213**	**23.6**	**20.8, 26.4**	**351**	**39.0**	**36.2, 41.8**
*OB/Gyn & Subspecialties*												
Manitoba & Saskatchewan	33	56.9	44.2, 69.6	20	60.6	43.9, 77.3	5	20.0	4.3, 35.7	9	33.3	15.5, 51.1
Atlantic	35	50.0	38.3, 61.7	24	68.6	53.2, 84.0	4	11.4	0.9, 21.9	6	20.7	6.0, 35.4
Alberta & British Columbia	93	68.4	60.6, 76.2	70	75.3	66.5, 84.1	17	39.5	24.9, 54.1	36	48.0	36.7, 59.3
Ontario	208	77.0	72.0, 82.0*	137	65.9	59.5, 72.3	11	17.7	8.2, 27.2	33	40.7	30.0, 51.4
Quebec	167	75.9	70.2, 81.6*	116	69.5	62.5, 76.5	17	32.1	19.5, 44.7	1	16.7	−13.1, 46.5
**OB/Gyn & Subspecialties Avg.**	**536**	**71.1**	**59.4, 82.8**	**367**	**68.6**	**53.2, 84.0**	**54**	**24.8**	**19.1, 30.5**	**85**	**39.0**	**32.5, 45.5**
*Paediatrics & Subspecialties*												
Manitoba & Saskatchewan	39	38.6	29.1, 48.1**	25	64.1	49.0, 79.2	20	32.3	20.7, 43.9	9	26.5	11.7, 41.3
Atlantic	30	32.3	22.8, 41.8**	15	50.0	32.1, 67.9	20	31.7	20.2, 43.2	6	24.0	7.3, 40.7
Alberta & British Columbia	158	66.7	60.7, 72.7*	106	67.1	59.8, 74.4	24	30.4	20.3, 40.5	66	48.2	41.8, 54.6
Ontario	315	75.4	71.3, 79.5*	189	60.0	54.6, 65.4	17	16.5	9.3, 23.7	63	35.6	28.5, 42.7
Quebec	230	70.1	65.1, 75.1*	146	63.5	57.3, 69.7	23	23.5	15.1, 31.9	10	31.3	15.2, 47.4
**Paediatrics & Subspecialties Avg.**	**772**	**65.6**	**62.9, 68.3**	**481**	**62.3**	**58.8, 65.8**	**104**	**25.7**	**21.4, 30.0**	**154**	**38.0**	**33.3, 42.7**
*Psychiatry & Subspecialties*												
Manitoba & Saskatchewan	47	55.3	44.7, 65.9	38	68.1	61.8, 74.4	3	7.9	−0.7, 16.5	6	50.0	21.7, 78.3
Atlantic	36	51.4	39.7, 63.1	25	69.4	54.4, 84.4	2	5.9	−2.0, 13.8	10	29.4	14.1, 44.7
Alberta & British Columbia	207	68.8	63.6, 74.0*	141	80.9	69.7, 92.1*	26	27.7	18.7, 36.7	34	54.8	42.4, 67.2**
Ontario	329	85.0	81.4, 88.6*	236	71.7	66.8, 76.6**	11	19.0	8.9, 29.1	77	52.4	44.3, 60.5**
Quebec	300	84.7	81.0, 88.4*	214	71.3	66.2, 76.4**	18	33.3	20.7, 45.9	6	26.1	8.2, 44.0
**Psychiatry & Subspecialties Avg.**	**919**	**76.8**	**74.4, 79.2**	**654**	**71.2**	**68.3, 74.1**	**60**	**21.6**	**16.8, 26.4**	**133**	**47.8**	**32.5, 63.1**
*Surgical Specialties (excl. OB/Gyn)*												
Manitoba & Saskatchewan	67	32.8	26.4, 39.2	30	44.8	32.9, 56.7	46	33.6	25.7, 41.5	16	24.2	13.9, 34.5
Atlantic	63	29.9	23.7, 36.1	34	54.0	41.7, 66.3	40	27.0	19.8, 34.2	14	20.3	10.8, 29.8
Alberta & British Columbia	316	59.5	55.3, 63.7*	219	69.3	64.2, 74.4	78	36.3	29.9, 42.7	106	34.4	29.1, 39.7
Ontario	793	77.1	74.5, 79.7*	473	59.6	56.2, 63.0	72	30.6	24.7, 36.5	113	25.2	21.2, 29.2
Quebec	584	72.5	69.4, 75.6*	402	68.8	65.0, 72.6	84	38.0	31.6, 44.4	19	29.2	18.1, 40.3
**Surgical Specialties** **(excl. OB/Gyn) Avg.**	**1,823**	**65.6**	**63.8, 67.4**	**1,158**	**63.5**	**61.3, 65.7**	**320**	**33.5**	**30.5, 36.5**	**268**	**28.0**	**25.1, 30.9**

a.Refers to the retention proportion of graduates who remained in the same service region group for both undergraduate and postgraduate medical education training.

b.Refers to the retention proportion of graduates who completed both their undergraduate and postgraduate training in the same service and remained in the same service region group 5-years into professional practice.

c.Refers to the retention proportion of graduates who completed their undergraduate and postgraduate training in different service region groups, and returned to the service region group where they completed their undergraduate medical training 5-years into professional practice.

d.Refers to the retention proportion of graduates who remained in the same service region 5-years into professional practice where they completed their postgraduate training.

e.CI (confidence interval) indicates the range within which we can be 95% confident that the true retention proportion lies. The narrower the CI, the more precise the estimate.

For example, a retention rate of 48.3 and 95% CI of 45.7, 50.9** means that the true retention rate is likely between 45.7% and 50.9%.

Significance levels (*p < 0.05, **p < 0.001) indicate the statistical significance of the results compared to the national average retention rate, with lower p-values representing stronger evidence against the null hypothesis.

Overall, 76.3% of all graduates completed both undergraduate and postgraduate medical training in the same service region group. The Quebec (86.5%) (possibly due to language) and Ontario (80.4%) service region groups had above-average retention rates across the medical education training continuum. However, retention rates dropped to 67.2% for medical graduates who remained in the same service region group where they completed both their undergraduate and postgraduate training five years into professional practice. Furthermore, one-quarter (24.9%) of all graduates practiced in the same service region group where they completed their undergraduate medical training, with the Alberta & British Columbia (32.7%) and Quebec (26.1%) service region groups demonstrating higher-than-average retention rates. Moreover, approximately 40% of all graduates five years into practice remained in the same service region group where they completed their residency, with the Alberta & British Columbia service region group having higher-than-average retention rates (48.7%).

Retention patterns also varied across medical specialties. Family medicine (81.9%) had the highest retention rates from undergraduate to postgraduate training, with the Quebec service region group retaining 93.4% of all family medicine graduates from undergraduate to postgraduate training, followed by the Ontario service region group (81.6%). Psychiatry (71.2%) had the highest retention rate across the training continuum and into professional practice, with the Alberta & British Columbia service region group retaining 81%, followed by the Ontario (71.7%), and Quebec (71.3%) service region groups. Surgical specialties had the highest retention from undergraduate medical education training to professional practice (33.5%), with the Quebec service region group retaining the highest proportion of graduates (38.0%), followed by the Alberta and British Columbia service region group (36.3%). Lastly, psychiatry had the highest retention rate from postgraduate training into professional practice (47.8%), with the Alberta & British Columbia service region group having the highest retention proportion (54.8%), followed by the Ontario (52.4%) service region group.

The Manitoba & Saskatchewan and Atlantic service region groups had lower retention rates than the national average across the training continuum and into professional practice and medical specialties. However, these service region groups showed above-average retention rates for certain medical specialties. Specifically, in paediatrics, the Atlantic (31.7%) and Manitoba & Saskatchewan (32.3%) service region groups had higher retention rates than the national average from undergraduate medical education into professional practice. Additionally, the Manitoba & Saskatchewan service region group demonstrated a higher-than-average retention rate (50.0%) in psychiatry from postgraduate training into professional practice. On the other hand, the Alberta & British Columbia service region group had high retention rates across the training continuum into professional practice and medical specialties, except for retention from undergraduate to postgraduate medical education.

## Discussion

This study introduces a methodological approach for creating medical school service regions and analyzes in-region graduate retention patterns by medical specialty across the medical education training continuum and into professional practice. It highlights the importance of considering medical specialty and practice location when planning and retaining the physician workforce. Additionally, this study offers important insights into social accountability, shedding light on the extent to which schools are able to retain graduates. However, it is important to acknowledge that each medical school contributes to graduate retention to varying degrees. While all Canadian medical schools adhere to rigorous accreditation standards set forth by the AFMC [63], ensuring comparability in quality and content, each school operates autonomously in its admissions processes, pedagogical approach, assessment strategies, and social accountability mandate. These differences may impact graduate retention and reflect the varying extents to which social accountability is implemented in their programs. For instance, some schools may have targeted admission pathways for rural and underserved areas, robust community-based training programs, and curricula focused on primary care, which are known to positively influence graduate retention in local regions. Additionally, some service region groups may have external programs or initiatives that could influence retention rates, such as government policies, community health initiatives, financial incentives, and other support mechanisms [4,64–66]. These differences highlight the need for further research to evaluate specific policies and practices of individual schools as well as external factors that may impact graduate retention rates.

Findings from this study underscore the need of understand graduate retention patterns to advance social accountability in medical education. Identifying service regions enables medical schools to gain a deeper understanding of their roles within specific areas and customize their programs to address local healthcare needs more effectively. Moreover, this process aligns with the social accountability mandate of medical schools, facilitating the evaluation of their success in retaining graduates within their designated regions and fulfilling their responsibility to serve the local community.

The findings reveal that certain service region groups were more successful than others in retaining graduates. Consistent with previous literature, our findings suggest that graduates tend to practice in the same region where they completed their medical training [16,32–34,67]. However, attending both undergraduate and postgraduate medical education in the same region yielded higher professional practice retention proportions compared to postgraduate training alone. Conversely, the location of postgraduate training produced higher in service region group practice retention proportions, compared to the proportion of graduates who returned to the service region group where they completed their undergraduate medical training [68]. This finding may be of particular concern for smaller schools that offer limited postgraduate training opportunities, as graduates may need to obtain specialized training elsewhere, making them less likely to return to practice where they completed their undergraduate medical training. However, service region groups with lower graduate retention rates may opt to implement local or rural admissions pathways and/or community-based training opportunities that have been shown to be associated with retaining graduates locally [5–13,25,26].

Although Canada’s graduate retention rate from postgraduate medical education into professional practice was slightly lower than what was reported in the United States in 2019 (54.6%) [69], the national retention proportion across the medical education continuum into professional practice was higher (67.2%). However, it is important to exercise caution when making country comparisons due to differences in data sources and sample characteristics. In contrast to previous literature reporting higher in-region retention rates for family medicine graduates [19,70], our findings suggest that psychiatry had higher retention rates across the medical training continuum into professional practice. Further research is warranted to examine the number of available psychiatry postgraduate training opportunities nationally and differences that may exist in residency selection.

Population size may also strongly influence the number of medical specialists who can be sustained in a particular service region [71–73]. This may be more extreme for traditional hospital-based specialties, as the population size of a given service region may strongly affect future practice locations. Our findings suggest that very few medical graduates in our sample practiced in the Territories, aligning with previous studies suggesting that lower retention rates are often observed in sparsely populated regions [6,64,67,68]. However, additional factors, such as graduates’ social-cultural considerations and the suitability of available opportunities may also contribute to the trend [64,68]. Further research is needed to examine the relative importance of a region’s population density, number of postgraduate training opportunities, and graduate demographics on retention patterns.

The study’s implications extend beyond education to health human resources and workforce planning, as policymakers can use graduate retention patterns to identify potential physician shortages and geographic maldistributions [72,73]. Perhaps one potential solution is for schools to create educational service regions. This study uses Canada as an example to demonstrate how schools can create geographic areas of primary responsibility likely to be served by each medical school using institutional websites and pre-existing administrative geographical boundaries. With service regions in place, schools can begin to better understand their primary regions of responsibility and identify areas of need relevant to their local context. While this study focused on domestic pathways, it is important to acknowledge the significant role international medical graduates (IMGs) in the Canadian healthcare system. IMGs represent a significant portion of the physician workforce in Canada, helping to address distribution issues across the country [74,75].

This paper provides a national overview of medical school service region groups and national graduate retention trends across 15 years of data. However, it is also important to acknowledge that graduate retention, specialty selection, and future practice location are multifaceted and influenced by a variety of personal, political, and economic factors beyond the control of medical schools [6]. Moreover, there are several other possible explanations for graduate retention that were not captured. These include hometown origin, socioeconomic status, rural or urban practice location, government policies, community health initiatives, financial incentives, and other support mechanisms [4,64–66]. Nonetheless, medical schools can contribute to their local communities by creating school service regions to better understand their primary regions of responsibility and identify areas of need [72]. Graduate retention may also be influenced by production-related factors, such as the availability of postgraduate training opportunities [64]. Therefore, it is important to consider the relationship between medical education, population health needs, and future capacity when determining production targets or the number of allocated training seats for health professionals [76,77]. Unfortunately, these relationships are seldom explored [71]. While graduate retention rates may not fully capture regional physician needs beyond location, they do provide a valuable measure of medical schools’ accountability to the communities they serve. However, it is important to acknowledge that retention rates are only one aspect of the complex factors that affect regional physician needs and healthcare delivery. Future research could further explore these factors and inform the development of more comprehensive strategies to meet the needs of underserved communities.

While graduate retention rates may not fully capture regional physician needs beyond location, they do provide a valuable measure of medical schools’ accountability to the communities they serve. However, it is important to acknowledge that retention rates are only one aspect of the complex factors that affect regional physician needs and healthcare delivery. Future research could further explore these factors and inform the development of more comprehensive strategies to meet the needs of underserved communities. Additionally, it is important to consider that some large urban schools have the capacity to train more students and offer more medical specialized postgraduate training programs than other smaller schools, independent of the population service need. Furthermore, certain medical specialized postgraduate programs only exist in some schools, necessitating a broader regional responsibility for those disciplines. For example, some schools may not have specialized medical postgraduate training programs, thus relying on other schools to provide specialists in a particular region.

Although the findings from this paper are not prescriptive, they can help policymakers better identify and predict physician specialty shortages and geographic maldistributions. One strategy for schools to encourage graduates to practice in their local communities is through local admissions pathways throughout the medical education continuum [76]. While the number of undergraduate and postgraduate medical education opportunities is often determined by national and local governments, medical schools could work collaboratively with governing bodies to ensure the appropriate number, mix, and distribution of physicians needed to meet community needs [1–3,72].

In addition, the use of pre-existing health region boundaries to identify medical school service regions may have limited our ability to examine local-level differences between population health needs. Nonetheless, health regions are continuously used by health authorities for planning, distribution, and allocation of health services and provide high-level understanding of population health needs and outcomes. Future research could examine graduate retention using smaller geographic units to better understand the individual contributions of each medical school to graduate retention and examine the impact of specific institutional policies and practices. Lastly, while this paper uses Canada as an example for creating service regions and examining graduate retention patterns, the methodology can be replicated in other contexts.

## Conclusion

This study created medical school service regions, examined graduate retention rates across the training continuum and into professional practice, and presented regional group comparisons and national benchmarks by medical specialty. Additionally, it highlights how schools can advance social accountability by examining the extent to which graduates practice within their respective service region group.

Findings from this study have important implications for medical schools, policymakers, and physician workforce planning. School service regions can be used to better track graduate outcomes and evaluate the effectiveness of rural and local admissions pathways, as well as extended community-based training, in fostering graduates who are more likely to practice in their local communities. Medical schools may consider their service region as a tool to better identify and serve local health needs., including monitoring and tracking graduate outputs to ensure that graduates and medical specialists are geographically dispersed [4,69]. Despite ongoing efforts by schools to improve graduate retention, the effectiveness of these initiatives remains largely unevaluated [37,78].

Poor graduate retention poses significant healthcare delivery challenges internationally, including persistent physician shortages and geographic and specialty maldistribution [65,68]. This study underscores the importance of creating educational service regions to examine in-region graduate retention. Further research is warranted to examine national in-region graduate retention trends, especially as health human resources continue to weigh heavily on policymakers [33]. From a policy perspective, fostering graduates into local practice requires national and local government support to secure adequate undergraduate and postgraduate opportunities across all medical specialties in respective regions [79]. Providing training opportunities across the medical education continuum seeks to further strengthen graduate retention [79]. Graduate retention represents an intermediate step to advancing health equity, serving as an outcome measure and pathway through which medical schools may contribute to social accountability and address societal needs.

This study advances social accountability in medical education by providing a methodology to examine graduate retention patterns. While aggregating school service region groups offers valuable insights, it is critical to consider the diverse initiatives and policies of individual schools that contribute to these patterns. Recognizing the varying degrees of school approaches to admissions, curricular, and assessment policies can help tailor strategies to improve graduate retention and address local healthcare needs more effectively.

## Supplementary Material

Supplemental Material

## Previous presentations

This paper was presented at the American Educational Research Association (AERA) annual meeting, April 2023, Chicago, Illinois, United States.
