# Supplementary material for: Medical school service regions in Canada: exploring graduate retention rates across the medical education training continuum and into professional practice
Source: Med Educ Online. 2024 Nov 3;29(1):2403805. doi: 10.1080/10872981.2024.2403805 (PMC11536692; doi:10.1080/10872981.2024.2403805)
Supplement: Supplemental Material [file ZMEO_A_2403805_SM0934.docx]

| **Supplemental Appendix I.**  Medical School Service Region Groups, Provinces and Territories, Medical School Service Regions (n=17) and 2018 Health Region Boundaries and Correspondence with Census Geography in Canada. | | | | |
| --- | --- | --- | --- | --- |
| **Medical School Service Region Groups** | **Province or Territory**^a^ | **Medical School Service Regions**^b^ | **Health Regions**^c^ | |
| Atlantic Region | Newfoundland & Labrador | Memorial University | Eastern Regional Health Authority | |
|  |  |  | Central Regional Health Authority | |
|  |  |  | Western Regional Health Authority | |
|  |  |  | Labrador-Grenfell Regional Health  Authority | |
|  | Prince Edward Island | Dalhousie University | Prince Edward Island | |
|  | Nova Scotia | Dalhousie University | Zone 1 - Western | |
|  |  |  | Zone 2 - Northern | |
|  |  |  | Zone 3 - Eastern | |
|  |  |  | Zone 4 - Central | |
|  | New Brunswick | Université de Sherbrooke | Zone 1 (Moncton area) | |
|  |  | Dalhousie University | Zone 2 (Saint John area) | |
|  |  |  | Zone 3 (Fredericton area) | |
|  |  |  | Zone 4 (Edmundston area  Zone 5 (Campbellton area) | |
|  |  |  | Zone 6 (Bathurust area)  Zone 7 (Miramichi area) | |
| Quebec Region | Quebec | Université Laval | Région du Bas-Saint-Laurent | |
|  |  |  | Région de al Capitale-Nationale | |
|  |  |  | Région du Chaudière-Appalaches | |
|  |  |  | Région de Laval | |
|  |  |  | Région de Lanaudière | |
|  |  |  | Région des Laurentides | |
|  |  |  | Région des Terres-Cries-de-la-  Baie-James | |
|  |  | Université de Sherbrooke | Région du Saguenay-Lac-Saint-  Jean | |
|  |  |  | Région de l'Estrie | |
|  |  |  | Région de al Gaspésie-Îles-de-la-  Madeleine | |
|  |  | Université de Montreal | Région de al Mauricie et du Centre-  du-Québec | |
|  |  |  | Région de Montréal | |
|  |  |  | Région de al Montérégie | |
|  |  | McGill University | Région de l'Outaouais | |
|  |  |  | Région de l'Abitibi-Témiscamingue | |
|  |  |  | Région de la Côte-Nord | |
|  |  |  | Région du Nord-du-Québec | |
|  |  |  | Région du Nunavik | |
| Ontario Region | Ontario (Public Health Regions) | McMaster University | Brant County Health Unit | |
|  |  |  | City of Hamilton Health Unit | |
|  |  |  | Niagara Regional Area Health Unit | |
|  |  |  | Waterloo Health Unit | |
|  |  | Northern Ontario School of Medicine | North Bay Parry Sound Health Unit | |
|  |  |  | Timiskaming Health Unit | |
|  |  |  | Northwestern Health Unit | |
|  |  |  | Porcupine Health Unit | |
|  |  |  | Renfrew County and District Health  Unit | |
|  |  |  | Sudbury and District Health Unit | |
|  |  |  | The District of Algoma Health Unit | |
|  |  |  | Thunder Bay District Health Unit | |
|  |  | University of Ottawa | City of Ottawa Health Unit | |
|  |  |  | Eastern Ontario Health Unit | |
|  |  |  | Haliburton, Kawartha, Pine Ridge  District Health Unit | |
|  |  |  | Leeds, Grenville and Lanark District  Health Unit | |
|  |  | Queen’s University | Hastings and Prince Edward  Counties Health Unit | |
|  |  |  | Kingston, Frontenac and Lennox  and Addington Health Unit | |
|  |  | University of Toronto | City of Toronto Health Unit | |
|  |  |  | Durham Regional Health Unit | |
|  |  |  | Halton Regional Health Unit | |
|  |  |  | Peel Regional Health Unit | |
|  |  |  | Peterborough County-City Health  Unit | |
|  |  |  | Simcoe Muskoka District Health  Unit | |
|  |  |  | Wellington-Dufferin-Guelph Health  Unit | |
|  |  |  | York Regional Health Unit | |
|  |  | Western University | Chatham-Kent Health Unit | |
|  |  |  | Grey Bruce Health Unit | |
|  |  |  | Haldimand-Norfolk Health Unit | |
|  |  |  | Huron County Health Unit | |
|  |  |  | Perth District Health Unit | |
|  |  |  | Lambton Health Unit | |
|  |  |  | Middlesex-London Health Unit | |
|  |  |  | Oxford Elgin St. Thomas Health  Unit | |
|  |  |  | Windsor-Essex County Health Unit | |
| Manitoba and Saskatchewan Region | Manitoba | University of Manitoba | Winnipeg Regional Health Authority | |
|  |  |  | Prairie Mountain Health | |
|  |  |  | Interlake-Eastern Regional Health  Authority | |
|  |  |  | Northern Regional Health Authority | |
|  |  |  | Southern Health | |
|  | Saskatchewan | University of Saskatchewan | Sun Country Regional Health  Authority | |
|  |  |  | Five Hills Regional Health Authority | |
|  |  |  | Cypress Regional Health Authority | |
|  |  |  | Regina Qu’Appelle Regional Health  Authority | |
|  |  |  | Sunrise Regional Health Authority | |
|  |  |  | Kelsey Trail Regional Health  Authority | |
|  |  |  | Saskatoon Regional Health  Authority | |
|  |  |  | Heartland Regional Health Authority | |
|  |  |  | Prairie North Regional Health | |
|  |  |  | Mamawetan Churchill River  Regional Health Authority | |
|  |  |  | Keewatin Yatthé Regional Health  Authority | |
|  |  |  | Athabasca Health Authority | |
|  |  |  | Mamawetan/Keewatin/Athabasca  Regional Health Authorities | |
|  |  |  | Prince Albert Parkland Regional  Health Authority | |
| Alberta & British Columbia Region | Alberta | University of Calgary | South Zone | |
|  |  |  | Calgary Zone | |
|  |  |  | Central Zone | |
|  |  | University of Alberta | Edmonton Zone | |
|  |  |  | North Zone | |
|  | British Columbia | The University of British Columbia | East Kootenay Health Service  Delivery Area | |
|  |  |  | Kootenay-Boundary Health Service  Delivery Area | |
|  |  |  | Okanagan Health Service Delivery  Area | |
|  |  |  | Thompson/Cariboo Health Service  Delivery Area | |
|  |  |  | Fraser East Health Service Delivery  Area | |
|  |  |  | Fraser North Health Service Delivery  Area | |
|  |  |  | Fraser South Health Service  Delivery Area | |
|  |  |  | Richmond Health Service Delivery  Area | |
|  |  |  | Vancouver Health Service Delivery  Area | |
|  |  |  | North Shore/Coast Garibaldi Health  Service Delivery Area | |
|  |  |  | South Vancouver Island Health  Service Delivery Area | |
|  |  |  | Central Vancouver Island Health  Service Delivery Area | |
|  |  |  | North Vancouver Island Health  Service Delivery Area | |
|  |  |  | Northwest Health Service Delivery  Area | |
|  |  |  | Northern Interior Health Service  Delivery Area | |
|  |  |  | Northeast Health Service Delivery  Area | |
| Territories | Yukon |  | Yukon | |
|  | Northwest Territories | | | Northwest Territories |
|  | Nunavut |  | Nunavut | |
| Notes: ^a^ There are 10 provinces and 3 Territories in Canada.  ^b^ Medical School Service Regions refer to geographically sensitive areas schools have a responsibility to serve.  ^c^ Health Regions refer to administrative areas defined by the provincial ministries of health and are used to make health care decisions. | | | | |
